# Effect that an educational program for cystic fibrosis patients and caregivers has on the contamination of home nebulizers*

**DOI:** 10.1590/S1806-37132014000200004

**Published:** 2014

**Authors:** Adriana Della Zuana, Doroti de Oliveira Garcia, Regina Célia Turola Passos Juliani, Luiz Vicente Ribeiro Ferreira da Silva

**Affiliations:** Graduate Program in Health Sciences, University of São Paulo School of Medicine, São Paulo, Brazil; Bacteriology Center, Adolfo Lutz Institute, São Paulo, Brazil; Hospital Division, Physical Therapy Section, Professor Pedro de Alcântara Institute for Children, University of São Paulo School of Medicine Hospital das Clínicas, São Paulo, Brazil; Department of Pulmonology, Professor Pedro de Alcântara Institute for Children, University of São Paulo School of Medicine Hospital das Clínicas, São Paulo, Brazil

**Keywords:** Cystic fibrosis, Nebulizers and vaporizers, Disinfection

## Abstract

**OBJECTIVE:**

To describe the pathogens found in home nebulizers and in respiratory samples of cystic fibrosis (CF) patients, and to evaluate the effect that a standardized instruction regarding cleaning and disinfection of nebulizers has on the frequency of nebulizer contamination.

**METHODS:**

We included 40 CF patients (22 males), all of whom used the same model of nebulizer. The median patient age was 11.2 ± 3.74 years. We collected samples from the nebulizer mouthpiece and cup, using a sterile swab moistened with sterile saline. Respiratory samples were collected by asking patients to expectorate into a sterile container or with oropharyngeal swabs after cough stimulation. Cultures were performed on selective media, and bacteria were identified by classical biochemical tests. Patients received oral and written instructions regarding the cleaning and disinfection of nebulizers. All determinations were repeated an average of two months later.

**RESULTS:**

Contamination of the nebulizer (any part) was detected in 23 cases (57.5%). The nebulizer mouthpiece and cup were found to be contaminated in 16 (40.0%) and 19 (47.5%), respectively. After the standardized instruction had been given, there was a significant decrease in the proportion of contaminated nebulizers (43.5%).

**CONCLUSIONS:**

In our sample of CF patients, nebulizer contamination was common, indicating the need for improvement in patient practices regarding the cleaning and disinfection of their nebulizers. A one-time educational intervention could have a significant positive impact.

## Introduction

Cystic fibrosis (CF) patients are highly susceptible to colonization by and lung infection with specific bacteria, and the establishment of a chronic bronchopulmonary infection is the leading cause of progressive lung injury.^(^ ^1^ ^)^ The increasingly frequent need for prescribing inhaled medications to these patients has led to greater use of home nebulizers.^(^ ^2^ ^,^ ^3^ ^)^ It is accepted that pathogens are commonly isolated from nebulizers, and there is a concern that nebulizer equipment may be a contributing source of bacterial infection into the lower airways of these patients.^(^ ^2^ ^,^ ^4^ ^)^

According to Rosenfeld et al.,^(^ ^5^ ^)^ hospitals have developed strict protocols for sterilization of nebulizers. In contrast, there are no guidelines for cleaning home nebulizers or existing guidelines are not well established.^(^ ^6^ ^,^ ^7^ ^)^ Hutchinson et al.^(^ ^8^ ^)^ suggest that contamination of home nebulizers is common and that it may be due to the variety of maintenance practices. Vassal et al.^(^ ^9^ ^)^ emphasize that, in the absence of cleaning, most nebulizers of CF patients are contaminated with a pathogenic flora.

The risk of contamination of home nebulizer equipment depends on various factors, such as the type of equipment used, including the material the nebulizer is made of; the efficiency of the cleaning and disinfection method recommended to patients; the microbiological quality of tap water (if used); and the quality of patient adherence to recommendations.^(^ ^10^ ^)^ In addition, Jakobsson et al.^(^ ^11^ ^)^ are convinced that oral and written instructions given to patients and their caregivers regarding nebulizer cleaning and disinfection practices are important for maintaining high levels of adherence to these practices.

In 2003, a consensus statement on the importance of infection control in CF developed by the Cystic Fibrosis Foundation (CFF) mentioned proper cleaning and disinfection of home nebulizers as one of the relevant principles.^(^ ^12^ ^)^ In addition, that study pointed out the need for continuing educational programs so that good levels of adherence can be achieved.^(^ ^12^ ^)^

The objective of the present study was to describe the pathogens found in home nebulizers of and in respiratory samples from CF patients, and to evaluate the effect that a standardized instruction regarding cleaning and disinfection of nebulizers has on the frequency of nebulizer contamination.

## Methods

The study sample consisted of patients diagnosed with CF, in accordance with international standards,^(^ ^13^ ^)^ who were being treated at the Pediatric Pulmonology Outpatient Clinic of the University of São Paulo School of Medicine *Hospital das Clínicas* Institute for Children, located in the city of São Paulo, Brazil. Patients were selected on the basis of the following inclusion criteria: using a PRONEB^(r)^ nebulizer and compressor system (PARI Medical Holding GmbH, Starnberg, Germany) and indicating interest in participating in the study upon receiving a telephone call. During a routine hospital visit, the parents or legal guardians of the patients received information about the study and gave written informed consent.

At the study outset, patients were instructed to bring the entire nebulizer system for verification. There was no mention of it being an assessment of contamination. A questionnaire was administered to establish what home method for cleaning and disinfecting nebulizers had been used until then.

At the time, samples were collected from the nebulizer medicine cup and mouthpiece for microbiological culture, by swabbing of the inner surface of the nebulizer medicine cup and mouthpiece with a sterile swab moistened with sterile saline (rotating the swab ten times clockwise).^(^ ^14^ ^)^

In addition, sputum samples or oropharyngeal swabs were collected from patients for microbiological culture. Sputum was collected by asking patients to expectorate into a sterile container, and oropharyngeal swabs were collected by rubbing of the retropharynx and pharyngeal pillars with a sterile swab (BD Brasil, São Paulo, Brazil). The samples collected from the patients and from the nebulizers, all of which were properly identified, were placed into an insulated bag with ice packs and sent to the microbiology laboratory within a maximum of three hours.

Cultures were performed at the Bacteriology Laboratory of the Adolfo Lutz Institute, located in the city of São Paulo, Brazil. The sputum samples and the oropharyngeal samples were directly smeared onto selective media. The media used included chocolate Agar, MacConkey agar, and selective media for the *Burkholderia cepacia* complex (B. cepacia selective medium; Oxoid Ltd., Basingstoke, UK), *Stenotrophomonas maltophilia*, and *Staphylococcus aureus*-Baird-Parker agar and/or mannitol agar (Oxoid)-and all cultures were incubated at 37°C for 16-72 h.^(^ ^15^ ^)^

The gram-negative bacilli isolated were identified phenotypically by extensive conventional biochemical tests that are already part of the routine practice of the Adolfo Lutz Institute.

The adopted cleaning and disinfection instructions were adapted from the model recommended by the CFF^(^ ^12^ ^)^ and from the instructions provided by the manufacturer of the nebulizer system used by the patients. At the end of sample collection, each patient and/or guardian received oral and written instructions regarding a standardized cleaning and disinfection process to be used henceforth that consisted of the following steps:

1. Cleaning: after use, the nebulizer should be disassembled and its parts should be washed inside and outside with mild detergent and tap water (except for the hose and its adapter, which should remain connected to the compressor for two minutes or should be left with the two ends hanging down in order to dry) and should be rinsed with tap water.
2. Disinfection: place the disassembled parts into a container filled with water and let it boil for five minutes. If the parts are disinfected with boiling water, rinsing is not necessary. Do not boil the hose, its adapter, or the mask. Repeat this procedure once a day.
3. Drying: after the final rinse, let the water drain from the material and dry it preferably with paper towels or a clean cloth.
4. Storage: assemble all parts of the nebulizer and store it in a container used for that sole purpose.

Patients were asked to bring their nebulizer equipment again at the next medical visit, and additional samples were collected from the nebulizers and the patients. At the time, the questionnaire was readministered in order to determine adherence to the recommended standardized method.

The study project was approved by the ethics committees of the Institute for Children and the Adolfo Lutz Institute, as well as by the Research Ethics Committee of the University of São Paulo School of Medicine *Hospital das Clínicas* (Protocol no. 0067/08).

For the purposes of the statistical analysis, categorical variables are expressed as frequencies and confidence intervals, and continuous variables are expressed as means, standard deviations, medians, and maximum and minimum values. The association between positive cultures and the remaining categorical variables was investigated by Fisher's exact test or the chi-square test. The difference between the frequencies of nebulizer contamination before and after the cleaning instructions had been given was assessed by McNemar's test. To determine whether the time interval between the first and second assessments would affect the results, we used a generalized estimating equations statistical model with binomial distribution,^(^ ^16^ ^)^ considering the time interval between the two assessments as a covariate. The sample size was calculated to yield a power of 80% to detect a 50% decrease in the frequency of nebulizer contamination, considering that, according to data in the literature,^(^ ^14^ ^)^ the rate of nebulizer contamination would be approximately 65% before the application of the proposed technique. For all calculations, the level of significance was set at < 5%. Statistical analyses were performed with PASW Statistics 18 (IBM Corp., Armonk, NY, USA).

The research project was funded entirely by the department and laboratories involved. Interviews and sample collection were performed at the Physical Therapy Outpatient Clinic of the Institute for Children by the principal researcher.

## Results

We evaluated 40 CF patients (22 males and 18 females) aged 5 to 18 years (median, 11.2 years). Among the 40 patients evaluated, all (100%) were being treated with inhaled DNase (Pulmozyme^(r)^; Roche, São Paulo, Brazil) and 16 (40%) were receiving inhaled antibiotic concomitantly. The median time between the evaluations was 63 days (range, 3-203 days).

**Figure 1:**
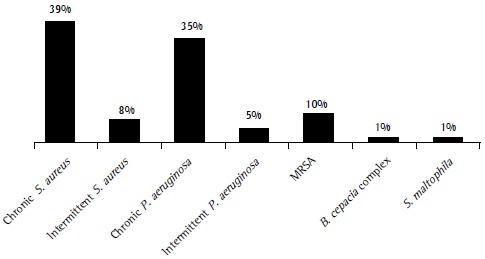
Prior colonization of the patients included in the study (n = 40). S. aureus: Staphylococcus aureus; P. aeruginosa: Pseudomonas aeruginosa; B. cepacia: Burkholderia cepacia; MRSA: methicillin-resistant S. aureus; and S. maltophilia: Stenotrophomonas maltophilia.

The colonization profile of the patients, which was obtained through analysis of medical records, showed a predominance of chronic colonization with *S. aureus* and *Pseudomonas aeruginosa* and a lower frequency of colonization with the *B. cepacia* complex and *S. maltophilia* (Figure 1).

The data obtained from the questionnaire administered to assess patient practices regarding the cleaning and disinfection of their nebulizers showed that, at the time of the first collection, 16 patients (40%) reported having already received instruction on such practices from a professional. Approximately 80% of the patients reported being aware of the importance of proper cleaning, but only 11 (27.5%) considered their cleaning and disinfection practices satisfactory. Patient practices regarding the cleaning, disinfection, drying, and storage of their nebulizer equipment varied widely, and most were considered unsatisfactory; however, there was a marked change after the instructions had been given (Table 1).

**Table 1:** Report of study participants' (n = 40) practices regarding the cleaning, disinfection, drying, and storage of their home nebulizers at the two questionnaire administrations.a

Question	First administration	Second administration
You have been instructed on how to clean and disinfect your nebulizer	16 (40.0)	40 (100.0)
The instruction was given by		
A physician	3 (7.5)	0 (0.0)
A nurse	0 (0.0)	0 (0.0)
A physical therapist	8 (20.0)	40 (100.0)
Others	5 (12.5)	0 (0.0)
You are aware of the importance of proper cleaning	32 (80.0)	38 (95.0)
You consider the way you clean your nebulizer equipment		
Satisfactory	11 (27.5)	36 (90.0)
Marginally satisfactory	19 (47.5)	3 (7.5)
Unsatisfactory	0 (0.0)	0 (0.0)
Do not know	10 (25.0)	1 (2.5)
Number of uses per day		
1	27 (67.5)	29 (72.5)
2	0 (0.0)	2 (5.0)
> 2	13 (32.5)	9 (22.5)
Frequency of cleaning per week		
1	1 (2.5)	0 (0.0)
2	3 (7.5)	0 (0.0)
3	1 (2.5)	0 (0.0)
7	10 (25.0)	1 (2.5)
After each inhalation	22 (55.0)	39 (97.5)
Parts that are cleaned		
Cap	40 (100.0)	40 (100.0)
Cup	39 (97.5)	40 (100.0)
Mouthpiece	40 (100.0)	40 (100.0)
Mask	0 (0.0)	0 (0.0)
Inner supply tube	39 (97.5)	40 (100.0)
Hose	29 (72.5)	31 (77.5)
Compressor	24 (60.0)	24 (60.0)
How you clean your nebulizer		
Disassemble it into parts	39 (97.5)	40 (100.0)
Scrubbing with your hands	15 (37.5)	24 (60.0)
Scrubbing with a sponge	17 (42.5)	14 (35.0)
Scrubbing with a cloth	0 (0.0)	1 (2.5)
Detergent	27 (67.6)	40 (100.0)
Tap water	33 (82.5)	40 (100.0)
Boiled water	5 (12.5)	0 (0.0)
Rinsing	32 (80.0)	40 (100.0)
How you disinfect your nebulizer		
Alcohol	3 (7.5)	1 (2.5)
Another product	1 (2.5)	0 (0.0)
Soaking	26 (65.0)	1 (2.5)
Rinsing with hot water	8 (20.0)	1 (2.5)
Boiling of the parts	10 (25.0)	38 (95.0)
How you dry your nebulizer		
Natural air drying	22 (55.0)	8 (20.0)
Paper towel	8 (20.0)	22 (55.0)
Cloth	10 (25.0)	10 (25.0)
How you store your nebulizer		
Bag	7 (17.5)	8 (20.0)
Container	20 (50.0)	31 (77.5)
No specific storage place	13 (32.5)	1 (2.5)

a Values expressed as n (%)

Of the 80 respiratory secretion samples collected from the patients at the two assessments, 60 were sputum samples and 20 were oropharyngeal swabs. *S. aureus* predominated (in 68.75%), followed by *P. aeruginosa* (in 43.75%), the *B. cepacia* complex (in 3.75%), and *S. maltophilia* (in 2.75%).

**Figure 2:**
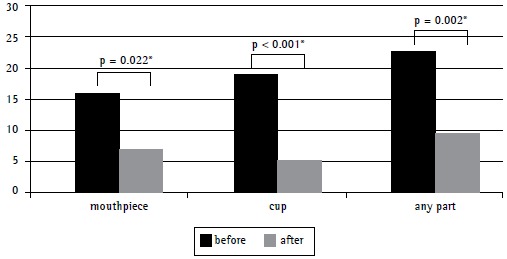
Frequency of nebulizer contamination before and after the standardized instruction (n = 40). *McNemar's test.

Contamination of the nebulizer (any part) was detected in 23 cases (57.5%), and contamination of the nebulizer mouthpiece and cup was detected in 16 and 19 cases, respectively (Table 2). After standardized instruction regarding the cleaning and disinfection of home nebulizers had been given, the number of contaminated nebulizer cases dropped to 10 (25%), and the number of contaminated nebulizer mouthpiece cases and contaminated nebulizer cup cases dropped to 7 and 5, respectively (Figure 2).

**Table 2:** Frequency of nebulizer contamination before and after the standardized instruction had been given (n = 40).

Nebulizer contamination	Before the instruction^a^	After the instruction^a^	p*	p after correction by the time interval between assessments**
Any part	23 (57.5)	10 (25.0)	0.002	0.001
Mouthpiece	16 (40.0)	7 (17.5)	0.022	0.011
Cup	19 (47.5)	5 (12.5)	< 0.001	< 0.001

a Values expressed as n (%)

* McNemar's test

** Generalized estimating equations model

The frequency of contamination decreased by 43.5%, which is significant considering the total number of contaminated nebulizers and the various parts of the nebulizer. However, the time interval between the two assessments had no influence on this decrease in contamination.

The nebulizer sample cultures detected a wide variety of microorganisms, with predominant detection of unidentified gram-negative bacilli (n = 14; Table 3). In 4 cases, the same microorganism was detected in the culture of the respiratory secretion sample from the patient and in the nebulizer (any part) sample culture. In 2 of those cases, the agent was identified as belonging to the genus *Pseudomonas*, and, in the other 2, it was identified as belonging to the genus *Staphylococcus*. Genetic analysis of these isolates (DNA macrorestriction analysis followed by pulsed-field gel electrophoresis) showed that they were unrelated strains (data not shown).

**Table 3:** Frequency of identification of microorganisms in the cultures of samples collected from the various parts of the nebulizers before and after the standardized instruction had been given (n = 40).

Microorganism	Before the instruction		After the instruction	
	Mouthpiece	Cup	Mouthpiece	Cup
Non-fermenting gram-negative bacilli	2	8	1	3
Coagulase-negative* Staphylococcus* sp.	6	2	3	2
*Acinetobacter* sp.	2	3	3	2
Yeasts	8	4	0	0
*Pseudomonas* putida	1	6	0	0
*Enterobacter* spp.	1	3	0	1
*Enterobacter*ia spp.	2	3	0	0
*Klebsiella* sp.	0	2	1	1
*Stenotrophomonas maltophilia*	1	1	0	1
Gram-positive bacilli	1	2	0	0
*Burkolderia cepacia complex*	1	1	0	1
*P. fluorescens*	1	2	0	0
*P. aeruginosa*	1	2	0	0
*Escherichia coli*	0	1	1	0
*S. aureus*	1	0	1	0
*Achromobacter xylosoxidans*	0	1	0	0

## Discussion

Most CF patients use nebulizers routinely,^(^ ^2^ ^)^ and, in the present study, the prevalence of contamination of home nebulizers was found to be quite significant (57.5%), despite the fact that most patients reported being aware of the importance of nebulizer cleaning and disinfection practices. This indicates the need for improvement in these practices.

The nebulizer cleaning and disinfection methods reported by patients before the standardized instruction had been given were, in most cases, not in line with international recommendations^(^ ^12^ ^)^, and only 25% of patients boiled the nebulizer parts, which is recommended by the CFF as a disinfection method.

Patients having received one-time standardized oral and written instructions resulted in a 43.5% decrease in contamination within an average of two months between the two assessments, which shows the potential of educational interventions in such a scenario.

Vassal et al.^(^ ^9^ ^)^ conducted a study in which 44 patients had chronic colonization with *P. aeruginosa*, 30 of whom (68%) had a nebulizer that had been contaminated with bacteria immediately after drug nebulization and did not receive any cleaning. Comparatively, the rate of nebulizer contamination found in the present study was 57.5%. Likewise, Blau et al.,^(^ ^14^ ^)^ in a study on bacterial contamination of nebulizers in the home treatment of CF patients, evaluated 29 nebulizer systems and found contamination in 19 (65%), *P. aeruginosa* being identified in 10 (35%). In contrast, in a study conducted in Brazil by Brzezinski et al.,^(^ ^3^ ^)^ only 6 (21%) of 28 nebulizers evaluated were contaminated with bacteria related to CF. The main difference between that study and ours is that, in the former, sample collection occurred at home visits, and it is of note that the samples were left at room temperature before being taken for analysis.^(^ ^3^ ^)^

Although in the present study we found a relatively small proportion of microorganisms typical of CF in the nebulizer sample cultures, a significant proportion of these cultures (n = 14) were found to be positive for non-fermenting gram-negative bacilli, which were not characterized phenotypically. These microorganisms can be pathogenic to CF patients, since there are relatively frequent reports of errors in microbiological identification.^(^ ^17^ ^,^ ^18^ ^)^

Rosenfeld et al.^(^ ^7^ ^)^ reported that the home nebulizer sample cultures from CF patients were frequently positive for *S. aureus* (55%), *P. aeruginosa* (35%), and species of the genus *Klebsiella* (19%). However, the concordance between sputum cultures and nebulizer sample cultures was poor. When studying 35 home nebulizers, Hutchinson et al.^(^ ^8^ ^)^ found that 3 were contaminated with the *B. cepacia* complex and 4 were contaminated with *S. maltophilia*. Although 34 patients had *P. aeruginosa* in their sputum, none of the nebulizers were positive for this microorganism. In addition, those authors reported that, even after cleaning, 69% of the nebulizers were contaminated with various types of gram-negative bacteria.

Blau et al.^(^ ^14^ ^)^ stated that the manufacturer's instructions provided with PARI Medical Holding GmbH nebulizer systems were inadequate, since they still recommended soaking the nebulizer in a solution of water and acetic acid for disinfection, which does not ensure disinfection against *S. aureus *or* B. cepacia*.^(^ ^2^ ^)^ Instructions currently available on that manufacturer's website have been updated in accordance with the CFF recommendations.^(^ ^19^ ^)^ In addition, Reychler et al.^(^ ^2^ ^)^ reported no benefits of drying; however, they recognize that this recommendation should be taken into account because pathogens such as *P. aeruginosa* and *B. cepacia* are hydrophilic, and drying should be a step in the cleaning process. The consensus statement published by the CFF^(^ ^12^ ^)^ states that the practices regarding the cleaning, disinfection, and drying of nebulizer parts are key steps for infection control in CF patients, both at home and in the hospital setting. However, data from questionnaires administered to CF patients regarding their home nebulizer cleaning and disinfection routine show a wide variety of cleaning practices.^(^ ^20^ ^)^ At our facility, the recommendations regarding the cleaning and disinfection of nebulizers used to be made in an empirical (non-standardized) way; after the results of the present study were made known, the CFF recommendations were adopted. This one-time educational intervention delivered orally and in writing by the same professional resulted in a significant decrease in contamination of the nebulizer equipment, despite the varying time interval between assessments.

The development of recommendations, such as those by the CFF, is only the first step in infection control; it is necessary to disseminate information and educate patients and their caregivers about cleaning and disinfection practices, since there may be cultural and social barriers to their implementation.^(^ ^21^ ^,^ ^22^ ^)^ In addition, education about these practices should be offered to undergraduate physical therapists and to all professionals who prescribe inhaled medications.^(^ ^4^ ^)^ Although various authors have recommended the use of oral and written instructions regarding these practices,^(^ ^10^ ^,^ ^11^ ^,^ ^14^ ^)^ our study unequivocally demonstrates the impact of this type of approach over an average two-month period of reassessment. However, among the limitations of the present study are the lack of a control group and the lack of subsequent sample collections to assess changes in the contamination profile of the nebulizer equipment, since it is possible that adherence to the recommended practices would decrease over time. Regarding the lack of a control group, we consider this to be an appropriate measure to minimize patient exposure to the theoretical risk of continuing to use contaminated nebulizer equipment, without being provided with correct instructions on how to clean and disinfect it at the first interview. Regarding the possibility of loss of effect, another assessment of contamination of the nebulizer equipment of the same patients would answer this query.

Future directions for studies in this area include determining more effective ways to promote adherence to infection control practices and developing mechanisms to assess the clinical impact of these practices on the basis of the results obtained with patients.
