# Supplementary material for: Effect that an educational program for cystic fibrosis patients and caregivers has on the contamination of home nebulizers
Source: J Bras Pneumol. 2014 Mar-Apr;40(2):119–27. doi: 10.1590/S1806-37132014000200004 (PMC4083641; doi:10.1590/S1806-37132014000200004)
Supplement: Translation

# Translation: Efeito de um programa de educação para cuidadores e pacientes com fibrose cística na contaminação de nebulizadores de uso domiciliar^*^

**Authors:** Adriana Della Zuana, Doroti de Oliveira Garcia, Regina Célia Turola Passos Juliani, Luiz Vicente Ribeiro Ferreira da Silva

**Affiliations:** Programa de Pós-Graduação em Ciências da Saúde, Faculdade de Medicina, Universidade de São Paulo, São Paulo (SP) Brasil; Centro de Bacteriologia, Instituto Adolfo Lutz, São Paulo (SP) Brasil; Divisão Hospitalar, Serviço de Fisioterapia, Instituto da Criança Professor Pedro de Alcântara, Hospital das Clínicas, Faculdade de Medicina, Universidade de São Paulo, São Paulo (SP) Brasil; Unidade de Pneumologia, Instituto da Criança Professor Pedro de Alcântara, Hospital das Clínicas, Faculdade de Medicina, Universidade de São Paulo, São Paulo (SP) Brasil

**Keywords:** Fibrose cística, Nebulizadores e vaporizadores, Desinfecção

## Abstract

**OBJETIVO:**

Descrever os patógenos encontrados nos nebulizadores de uso domiciliar e nas amostras de trato respiratório de pacientes com fibrose cística (FC) e verificar o efeito de uma instrução padronizada de higiene e desinfecção de nebulizadores na contaminação dos mesmos.

**MÉTODOS:**

Foram incluídos no estudo 40 pacientes com FC (22 do sexo masculino) que utilizavam um mesmo modelo de nebulizador. A mediana de idade foi de 11,2 ± 3,74 anos. Amostras dos nebulizadores foram coletadas do bocal e do copo reservatório utilizando-se um *swab* estéril umedecido em solução salina estéril. As amostras de trato respiratório dos pacientes foram colhidas por expectoração em coletor estéril ou com *swab* de orofaringe após estímulo de tosse. As culturas foram realizadas em meios seletivos, e a identificação bacteriana foi feita através de provas bioquímicas clássicas. Instruções verbais e escritas sobre higiene e desinfecção dos nebulizadores foram ministradas. Todas as determinações foram repetidas dois meses após, em média.

**RESULTADOS:**

A contaminação de alguma parte dos nebulizadores foi observada em 23 casos (57,5%). A contaminação do bocal e do copo foi similar, em 16 (40.0%) e 19 casos (47.5%), respectivamente. Houve uma redução significativa da proporção de nebulizadores contaminados (43,5%) após a instrução padronizada.

**CONCLUSÕES:**

Nesta amostra de pacientes com FC, a contaminação dos nebulizadores foi alta, o que indica a necessidade de melhoria nas práticas de higiene e desinfecção dos nebulizadores de pacientes com FC. Uma única intervenção educacional pode ter um impacto positivo significativo.

## Introdução

Os pacientes com fibrose cística (FC) apresentam uma grande suscetibilidade à colonização e infecção pulmonar por bactérias específicas, sendo o estabelecimento de uma infecção broncopulmonar crônica a maior causa de dano pulmonar progressivo.^(^ ^1^ ^)^ A necessidade de prescrição cada vez mais frequente de medicações por via inalatória para esses pacientes tem levado ao maior uso de nebulizadores para o uso domiciliar.^(^ ^2^ ^,^ ^3^ ^)^ É reconhecido que patógenos são comumente isolados nos nebulizadores, e existe uma preocupação de que esses equipamentos possam contribuir como uma fonte de infecção bacteriana para as vias aéreas inferiores nesses pacientes.^(^ ^2^ ^,^ ^4^ ^)^

De acordo com Rosenfeld et al.,^(^ ^5^ ^)^ os hospitais têm desenvolvido formas rigorosas para a esterilização dos nebulizadores. Em contrapartida, não há critérios para a sua limpeza domiciliar ou tais critérios não são bem estabelecidos.^(^ ^6^ ^,^ ^7^ ^)^ Hutchinson et al.^(^ ^8^ ^)^ sugerem que a contaminação de nebulizadores de uso domiciliar é frequente e que isso pode estar relacionado à variedade de práticas na manutenção dos mesmos. Vassal et al.^(^ ^9^ ^)^ destacam que, na ausência de limpeza, a maioria dos nebulizadores de pacientes com FC é contaminada por uma flora patogênica.

O risco de contaminação do equipamento domiciliar de inalação nos pacientes depende de vários fatores, tais como o tipo de equipamento usado, incluindo o tipo de material do nebulizador; a eficiência do método de limpeza e de desinfecção recomendada aos pacientes; a qualidade microbiológica da água de torneira (se usada); e a qualidade da adesão dos pacientes às recomendações.^(^ ^10^ ^)^ Jakobsson et al.^(^ ^11^ ^)^ estão convencidos ainda de que informações dadas verbalmente e por escrito aos pacientes e a seus cuidadores a respeito das práticas sobre limpeza e desinfecção dos nebulizadores são importantes para manter alta a adesão a essas práticas.

Em 2003, um consenso elaborado pela *Cystic Fibrosis Foundation *(CFF) sobre a importância do controle da infecção em FC cita como um dos princípios relevantes a higiene e a desinfecção apropriadas dos nebulizadores de uso domiciliar. ^(^ ^12^ ^)^ Aquela publicação indica ainda a necessidade de programas de educação continuada para a obtenção de bons níveis de adesão.^(^ ^12^ ^)^

O objetivo do presente estudo foi descrever os patógenos encontrados em nebulizadores de uso domiciliar e no trato respiratório de pacientes com FC e verificar a eficácia de uma técnica padronizada de higiene e desinfecção desses nebulizadores.

## Métodos

A casuística foi composta por pacientes com diagnóstico de FC estabelecido através de normas internacionais,^(^ ^13^ ^)^ acompanhados no Ambulatório de Pneumologia Pediátrica do Instituto da Criança, Hospital das Clínicas da Faculdade de Medicina da Universidade de São Paulo, localizado na cidade de São Paulo. Os pacientes foram selecionados de acordo com os seguintes critérios de inclusão: fazer uso de um sistema de compressor e nebulizador da marca PRONEB^(r)^ (PARI Medical Holding GmbH, Starnberg, Alemanha) e indicar interesse em participar da pesquisa através de um contato telefônico. Na visita periódica ao hospital, os pais ou responsáveis pelos pacientes receberam explicações sobre a pesquisa e assinaram o termo de consentimento informado.

Por ocasião do início do estudo, os pacientes foram instruídos a trazer o nebulizador completo para conferência. Não houve qualquer menção de que se tratava de uma avaliação da contaminação. Um questionário foi aplicado com a finalidade de conhecer o método domiciliar de higiene e desinfecção dos nebulizadores usado até então.

Naquela ocasião, foram coletadas amostras do copo reservatório da medicação e do bocal do nebulizador para a realização da cultura microbiológica mediante a seguinte técnica: um *swab* estéril umedecido em solução salina foi girado dez vezes em sentido horário na superfície interna tanto do copo reservatório da medicação quanto do bocal do nebulizador.^(^ ^14^ ^)^

Foram também coletados amostras de escarro ou esfregaço de orofaringe dos pacientes para a realização de cultura microbiológica. O escarro foi colhido através de expectoração em recipiente plástico estéril, e o esfregaço de orofaringe foi coletado através de fricção de um *swab* estéril (BD Brasil, São Paulo, Brasil) na retrofaringe e nos pilares faríngeos do paciente. As amostras de secreção do paciente e do nebulizador, devidamente identificadas, foram colocadas em uma bolsa térmica com gelo e encaminhadas para o laboratório de microbiologia num período máximo de três horas.

As culturas foram realizadas no Laboratório de Bacteriologia do Instituto Adolfo Lutz, localizado em São Paulo. Amostras de escarro e as amostras de orofaringe foram semeadas diretamente em meios seletivos. Foram utilizados ágar chocolate, ágar MacConkey e meios seletivos para o complexo Burkholderia cepacia (*B. cepacia selective medium*; Oxoid Ltd., Basingstoke, Reino Unido), *Stenotrophomonas maltophilia* e *Staphylococcus* aureus - agar Baird Parker e/ou agar manitol (Oxoid) - e incubadas a 37°C por 16-72 h.^(^ ^15^ ^)^

Os bacilos Gram-negativos isolados foram identificados fenotipicamente através de extensas provas bioquímicas convencionais já implantadas na rotina do Instituto Adolfo Lutz.

As orientações sobre higiene e desinfecção adotadas foram adaptadas do modelo recomendado pela CFF^(^ ^12^ ^)^ juntamente com orientações dadas pelo fabricante do sistema de nebulização utilizado pelos pacientes. Ao término da coleta, cada paciente e/ou responsável recebeu orientação verbal e escrita sobre o processo de higiene e desinfecção padronizado a ser obedecido doravante, que consistia nas seguintes etapas:

1. Higiene (limpeza): em seguida ao uso do nebulizador, ele deve ser desmontado, e suas partes devem ser lavadas por dentro e por fora com detergente neutro e água da torneira (exceto a mangueira e seu adaptador, que devem ser secos, deixando-os conectados por dois minutos no compressor ou com as duas extremidades penduradas para baixo) e enxaguadas com água da torneira.
2. Desinfecção: colocar as partes ainda desmontadas em um recipiente com água e deixar ferver por cinco minutos. Se forem desinfetadas com água fervente, não é necessário o enxágue. Não ferver a mangueira, seu adaptador ou a máscara. Realizar esse procedimento uma vez ao dia.
3. Secagem: após o enxágue final, deixar a água no material escoar e secá-lo preferencialmente com papéis toalhas ou pano limpo.
4. Armazenagem: montar todas as partes do nebulizador e guardar em um recipiente para essa única finalidade.

Foi solicitado aos pacientes que trouxessem seus equipamentos novamente na consulta médica seguinte, e novas coletas de material do nebulizador e do paciente foram realizadas. Naquela ocasião, o questionário foi reaplicado com a finalidade de conferir a adesão ao método padronizado orientado.

O projeto foi aprovado pelas comissões de ética do Instituto da Criança e do Instituto Adolfo Lutz, além da Comissão de Ética e Pesquisa do Hospital das Clínicas da Faculdade de Medicina da Universidade de São Paulo (CAPPesq no. 0067/08).

A análise estatística incluiu a descrição das variáveis categóricas, através de suas frequências e intervalos de confiança, e das variáveis contínuas, através de suas médias, desvios-padrão, medianas e valores máximos e mínimos. A associação entre a positividade das culturas e as demais variáveis categóricas foi investigada através do teste de Fisher ou do teste do qui-quadrado. A diferença entre a frequência de contaminação dos dispositivos antes e depois das orientações de higiene foi avaliada através do teste de McNemar. Para verificar se o tempo decorrido entre a primeira e a segunda avaliação influenciaria o resultado, utilizamos um modelo estatístico de equações generalizadas (*generalized estimating equations*) com distribuição binomial,^(^ ^16^ ^)^ considerando o tempo entre as duas avaliações como covariável. O tamanho da amostra foi calculado para ter um poder de 80% a fim de encontrar uma redução de 50% na frequência de contaminação dos nebulizadores, considerando-se, segundo dados na literatura,^(^ ^14^ ^)^ que haveria aproximadamente 65% de contaminação dos mesmos antes da aplicação da técnica proposta. Em todos os cálculos, foi adotado um nível de significância < 5%. As análises estatísticas foram realizadas com o programa PASW Statistics 18 (IBM Corp., Armonk, NY, EUA).

O projeto de pesquisa foi realizado com recursos próprios da unidade e dos laboratórios envolvidos. As entrevistas e as coletas foram realizadas no Ambulatório de Fisioterapia do Instituto da Criança pela pesquisadora principal.

## Resultados

Foram avaliados 40 pacientes com FC, sendo 22 do sexo masculino e 18 do sexo feminino, com idades entre 5 e 18 anos (mediana, 11,2 anos). Entre os 40 pacientes avaliados, todos (100%) faziam inalação com dornase alfa (Pulmozyme^(r)^; Roche, São Paulo, Brasil), sendo que 16 (40%) usavam algum tipo de antibiótico por via inalatória concomitantemente. A mediana de tempo entre as avaliações foi de 63 dias (variação, 3-203 dias).

O perfil de colonização dos pacientes, obtido através de análises de dados dos prontuários, mostrava um predomínio de colonização crônica por* S. aureus* e *Pseudomonas aeruginosa* e uma menor frequência de complexo *B. cepacia* e *S. maltophilia* (Figura 1).

**Figura 1:** Colonização prévia dos pacientes incluídos no estudo (n = 40). S. aureus: Staphylococcus aureus; P. aeruginosa: Pseudomonas aeruginosa; B. cepacia: Burkholderia cepacia; MRSA: methicillin-resistant S. aureus; e S. maltophilia: Stenotrophomonas maltophilia.

Os dados obtidos através do questionário aplicado para avaliar as práticas sobre higiene e desinfecção dos nebulizadores mostraram que, no momento da primeira coleta, 16 pacientes (40%) relatavam já terem sido orientados por algum profissional sobre tais práticas. Aproximadamente 80% dos pacientes relatavam conhecer a importância da limpeza, mas apenas 11 (27,5%) consideravam suas práticas de limpeza e desinfecção adequadas. As práticas de higiene, desinfecção, secagem e armazenagem dos equipamentos foram muito variáveis e, em sua maioria, consideradas inadequadas; contudo, observou-se uma grande alteração após as orientações ministradas (Tabela 1).

**Tabela 1:** Prática relatada dos participantes do estudo (n = 40) quanto a práticas de higiene, desinfecção, secagem e armazenagem dos nebulizadores de uso domiciliar nas duas aplicações do questionário utilizado.a

Questões	Primeira aplicação	Segunda aplicação
Já foi orientado anteriormente sobre a limpeza e desinfecção do nebulizador	16 (40,0)	40 (100,0)
A orientação foi dada por		
Médico	3 (7,5)	0 (0,0)
Enfermeiro	0 (0,0)	0 (0,0)
Fisioterapeuta	8 (20,0)	40 (100,0)
Outros	5 (12,5)	0 (0,0)
Conhece a importância da limpeza adequada do inalador	32 (80,0)	38 (95,0)
Considera a limpeza do seu equipamento de nebulização		
Adequada	11 (27,5)	36 (90,0)
Razoável	19 (47,5)	3 (7,5)
Inadequada	0 (0,0)	0 (0,0)
Não sabe	10 (25,0)	1 (2,5)
Número de utilizações por dia		
1	27 (67,5)	29 (72,5)
2	0 (0,0)	2 (5,0)
> 2	13 (32,5)	9 (22,5)
Frequência de limpeza na semana		
1	1 (2,5)	0 (0,0)
2	3 (7,5)	0 (0,0)
3	1 (2,5)	0 (0,0)
7	10 (25,0)	1 (2,5)
Depois de cada inalação	22 (55,0)	39 (97,5)
Quais as partes que são limpas?		
Tampa	40 (100,0)	40 (100,0)
Copo	39 (97,5)	40 (100,0)
Bocal	40 (100,0)	40 (100,0)
Máscara	0 (0,0)	0 (0,0)
Suplemento interno	39 (97,5)	40 (100,0)
Mangueira	29 (72,5)	31 (77,5)
Compressor	24 (60,0)	24 (60,0)
Como realiza a limpeza		
Desmonta em partes	39 (97,5)	40 (100,0)
Esfrega com as mãos	15 (37,5)	24 (60,0)
Esfrega com esponja	17 (42,5)	14 (35,0)
Esfrega com pano	0 (0,0)	1 (2,5)
Detergente	27 (67,6)	40 (100,0)
Água da torneira	33 (82,5)	40 (100,0)
Água fervida	5 (12,5)	0 (0,0)
Realiza enxágue	32 (80,0)	40 (100,0)
Como realiza a desinfecção		
Álcool	3 (7,5)	1 (2,5)
Outro produto	1 (2,5)	0 (0,0)
Deixa de molho	26 (65,0)	1 (2,5)
Água quente para enxaguar	8 (20,0)	1 (2,5)
Ferve as partes	10 (25,0)	38 (95,0)
Como realiza a secagem		
Naturalmente	22 (55,0)	8 (20,0)
Papel toalha	8 (20,0)	22 (55,0)
Pano	10 (25,0)	10 (25,0)
Como realiza a armazenagem		
Saco	7 (17,5)	8 (20,0)
Recipiente	20 (50,0)	31 (77,5)
Não tem local específico para a armazenagem	13 (32,5)	1 (2,5)

Dentre o total de 80 amostras coletadas de secreção respiratória dos pacientes nas duas avaliações, foram obtidas 60 amostras de escarro e 20 amostras de esfregaço de orofaringe. Houve um predomínio de* S. aureus* (68,75%), seguido de *P. aeruginosa *(43,75%), complexo *B. cepacia* (3,75%) e *S. maltophilia* (2,75%).

A contaminação dos nebulizadores por algum microrganismo foi observada em 23 casos (57,5%), sendo que a contaminação do bocal e do copo foi similar, com 16 e 19 casos, respectivamente (Tabela 2). Após a orientação padronizada de higiene e desinfecção dos nebulizadores de uso domiciliar, a proporção de nebulizadores contaminados caiu para 10 casos (25%), sendo a proporção de casos de contaminação em bocal e copo foram 7 e 5, respectivamente (Figura 2).

**Tabela 2:** Frequência de contaminação do nebulizador e de suas partes antes e depois da orientação padronizada (n = 40).

Tipo de contaminação	Contaminação antes das orientações^a^	Contaminação depois das orientações^a^	p*	p após correção pelo tempo entre as avaliações**
Qualquer tipo	23 (57,5)	10 (25,0)	0,002	0,001
Contaminação no bocal	16 (40,0)	7 (17,5)	0,022	0,011
Contaminação no copo	19 (47,5)	5 (12,5)	< 0,001	< 0,001

a Valores expressos em n (%)

* Teste de McNemar

** Modelo generalized estimating equations.

**Figura 2:** Frequência de contaminação dos nebulizadores antes e depois da orientação padronizada (n = 40). *Teste de McNemar.

A redução da frequência de contaminação foi de 43,5%, significativa para o total de nebulizadores contaminados e para as diferentes partes do nebulizador; entretanto não houve influência do tempo entre as duas avaliações no resultado da redução de contaminação.

As culturas dos nebulizadores identificaram uma grande diversidade de microrganismos, com predomínio de identificação de bacilos Gram-negativos sem identificação (n = 14; Tabela 3). Em 4 casos, houve a identificação de um mesmo microrganismo na cultura de secreção respiratória do paciente e em alguma parte do nebulizador; em 2 desses casos, o agente identificado foi do gênero *Pseudomonas* e, nos outros 2, foi do gênero *Staphylococcus*. A análise genética desses isolados (análise de macrorrestrição de DNA seguida de eletroforese em gel em campo pulsado) mostrou tratarem-se de cepas não relacionadas (dados não apresentados).

**Tabela 3:** Frequência de identificação de microrganismos nas culturas das diferentes partes dos nebulizadores antes e depois da orientação padronizada (n = 40).

Microrganismos	Antes da orientação		Depois da orientação	
	Bocal	Copo	Bocal	Copo
Bacilos Gram-negativos não fermentadores	2	8	1	3
*Staphylococcus* sp. coagulase negativo	6	2	3	2
*Acinetobacter* sp.	2	3	3	2
Leveduras	8	4	0	0
*Pseudomonas* putida	1	6	0	0
*Enterobacter* spp.	1	3	0	1
*Enterobacter*ia spp.	2	3	0	0
*Klebsiella* sp.	0	2	1	1
*Stenotrophomonas maltophilia*	1	1	0	1
Bacilos Gram-positivos	1	2	0	0
Complexo Burkolderia cepacia	1	1	0	1
*P. fluorescens*	1	2	0	0
*P. aeruginosa*	1	2	0	0
*Escherichia coli*	0	1	1	0
*S. aureus*	1	0	1	0
*Achromobacter xylosoxidans*	0	1	0	0

## Discussão

A maioria dos pacientes com FC faz uso de nebulizadores rotineiramente,^(^ ^2^ ^)^ e o presente estudo demonstrou uma prevalência de contaminação nos nebulizadores de uso domiciliar bastante significativa (57,5%), a despeito de a maioria dos pacientes relatar ter conhecimento da importância das práticas de limpeza e desinfecção dos mesmos, o que indica a necessidade de melhoria dessas práticas.

Os métodos de higiene e desinfecção dos nebulizadores referidos pelos pacientes antes da orientação padronizada estavam em desacordo com as recomendações internacionais^(^ ^12^ ^)^ na maioria dos casos, e somente 25% realizavam a fervura das partes, recomendada pela CFF como método de desinfecção.

A administração de uma única orientação padronizada verbal e por escrito a esses pacientes resultou numa redução da contaminação de 43,5% em um prazo médio de dois meses entre as avaliações, o que mostra o potencial de intervenções educativas em tal cenário.

Comparativamente em relação à taxa de contaminação nos nebulizadores encontrada no presente estudo (57,5%), Vassal et al.^(^ ^9^ ^)^ realizaram um estudo no qual 44 pacientes apresentavam colonização crônica por *P. aeruginosa*, sendo que 30 dos pacientes (68%) possuiam um nebulizador contaminado com algum tipo de bactéria logo após a nebulização do medicamento, sem fazer uso de nenhum tipo de higiene. Da mesma forma, Blau et al.,^(^ ^14^ ^)^ em um estudo sobre a contaminação bacteriana de nebulizadores no tratamento domiciliar de pacientes com FC, avaliaram 29 equipamentos e encontraram contaminação em 19 (65%), sendo identificada *P. aeruginosa *em 10 (35%). Em contrapartida, em um estudo realizado no Brasil e conduzido por Brzezinski et al.,^(^ ^3^ ^)^ apenas 6 nebulizadores de 28 avaliados (21%) estavam contaminados com bactérias relacionadas à FC. A principal diferença daquele estudo em relação ao nosso é que as coletas foram feitas em visita domiciliar, e vale ressaltar que o material coletado foi deixado em temperatura ambiente antes de ser levado para a análise.^(^ ^3^ ^)^

Apesar de no presente estudo termos encontrado uma proporção relativamente pequena de microrganismos típicos de FC nos nebulizadores, uma proporção significativa deles resultou na identificação de bacilos Gram-negativos não fermentadores (n = 14), que não tiveram uma caracterização fenotípica estabelecida. Esses microrganismos podem ser patogênicos para pacientes com FC, pois existem descrições de erros de identificação microbiológica de forma relativamente frequente.^(^ ^17^ ^,^ ^18^ ^)^

Rosenfeld et al.^(^ ^7^ ^)^ reportaram que as culturas de nebulizadores de uso domiciliar de pacientes com FC eram frequentemente positivas para* S. aureus* (55%), *P. aeruginosa *(35%) e espécies do gênero *Klebsiella* (19%). No entanto, a concordância entre as culturas do escarro e as dos nebulizadores foi pobre. Hutchinson et al.^(^ ^8^ ^)^ encontraram, ao estudar 35 nebulizadores de uso domiciliar, 3 contaminados com complexo *B. cepacia* e 4 contaminados com *S. maltophilia*. Apesar de 34 pacientes apresentarem *P. aeruginosa *no escarro, nenhum dos nebulizadores foi positivo para esse microrganismo. Os autores também demonstraram que mesmo após a limpeza 69% dos nebulizadores estavam contaminados por vários tipos de bactérias Gram-negativas.

Blau et al.^(^ ^14^ ^)^ afirmaram que as instruções do fabricante PARI Medical Holding GmbH para seus nebulizadores, que acompanham os equipamentos, eram instruções inadequadas, pois essas ainda sugeriam como método de desinfecção mergulhar o nebulizador em solução de água e ácido acético, o que não garante a desinfecção contra* S. aureus* e *B. cepacia*.^(^ ^2^ ^)^ Instruções atualmente disponíveis na página de Internet daquele fabricante foram atualizadas de acordo com as recomendações da CFF.^(^ ^19^ ^)^ Reychler et al.^(^ ^2^ ^)^ tampouco demonstraram benefícios na secagem; no entanto, reconhecem que essa recomendação deve ser levada em consideração visto que patógenos como *P. aeruginosa *e *B. cepacia* são hidrofílicos e a secagem deveria ser uma etapa do processo de higiene. O consenso publicado pela CFF^(^ ^12^ ^)^ declara que as práticas de higiene, desinfecção e secagem das peças dos nebulizadores são passos críticos no controle de infecção em pacientes com FC, tanto no domicílio como em ambientes hospitalares. Dados de questionários aplicados a pacientes com FC sobre a rotina de higiene e desinfecção dos nebulizadores de uso domiciliar mostram, entretanto, uma variedade diversa de práticas de higiene.^(^ ^20^ ^)^ Em nosso serviço, até então, a recomendação sobre a higiene e a desinfecção dos nebulizadores era feita de forma empírica (não padronizada) e, a partir do presente estudo, as recomendações da CFF foram adotadas. Essa única orientação ministrada por um mesmo profissional, de forma verbal e por escrito, resultou numa redução significativa da contaminação dos equipamentos, mesmo levando-se em conta o intervalo variável entre as avaliações.

O desenvolvimento de recomendações, como as da CFF, é apenas o primeiro passo no controle da infecção; é necessário disseminar as informações e educar os pacientes e seus cuidadores sobre essas práticas, pois podem existir barreiras culturais e sociais para a implementação das mesmas.^(^ ^21^ ^,^ ^22^ ^)^ Ainda, a educação sobre essas práticas deveria ser oferecida a fisioterapeutas durante sua formação universitária e a todos os profissionais que prescrevem medicações inalatórias.^(^ ^4^ ^)^ Apesar de vários autores recomendarem orientações verbais e por escrito nesse sentido,^(^ ^10^ ^,^ ^11^ ^,^ ^14^ ^)^ nosso estudo demonstra de forma inequívoca o impacto desse tipo de abordagem num período médio de dois meses de reavaliação. Entre as limitações de nosso estudo, entretanto, podemos citar a falta de um grupo controle e de coletas subsequentes para avaliar o cenário evolutivo da contaminação dos equipamentos, pois é possível que a adesão às práticas orientadas se reduzisse com o tempo. Em relação à falta de grupo controle, consideramos a atitude apropriada para minimizar a exposição dos pacientes ao risco teórico de continuarem com equipamentos contaminados, sem fornecer instruções corretas de como realizar sua higiene e descontaminação logo na primeira entrevista. Com relação à possibilidade da perda do efeito do estudo, uma nova avaliação da contaminação dos equipamentos desses mesmos pacientes poderia responder a esse questionamento.

As perspectivas futuras de estudos nessa área incluem determinar maneiras mais eficazes de promover a adesão às práticas de controle da infecção e o desenvolvimento de mecanismos para avaliar o impacto clínico dessas práticas através dos resultados com os pacientes.
